# A Genome-Wide Association Study Identifies Candidate SNPs and Genes Associated with Body Weight in the Northern Snakehead (*Channa argus*) Using a 50K SNP Array

**DOI:** 10.3390/ani16162471

**Published:** 2026-08-08

**Authors:** Haiyang Liu, Xinying Li, Wu Xie, Yu Wang, Mi Ou, Qing Luo, Shuzhan Fei, Xincheng Zhang, Weifeng Chen, Xinping Zhu, Kunci Chen, Jian Zhao

**Affiliations:** 1 Key Laboratory of Tropical and Subtropical Fishery Resources Application and Cultivation, Ministry of Agriculture and Rural Affairs, Pearl River Fisheries Research Institute, Chinese Academy of Fishery Sciences, Guangzhou 510380, China; hyliu@prfri.ac.cn (H.L.); 18245416908@163.com (X.L.); 18974014895@163.com (W.X.); wangy8957@gmail.com (Y.W.); om1990@prfri.ac.cn (M.O.); luoqing@prfri.ac.cn (Q.L.); feisz@prfri.ac.cn (S.F.); zhangxc@prfri.ac.cn (X.Z.); zhuxinping@prfri.ac.cn (X.Z.); chenkunci@prfri.ac.cn (K.C.); 2 Graduate School, Chinese Academy of Agricultural Sciences, Beijing 100081, China; 3 Jiangsu Key Laboratory for Eco-Agriculture Biotechnology Around Hongze Lake, Jiangsu Collaborative Innovation Center of Regional Modern Agriculture & Environmental Protection, Jiangsu Engineering Laboratory for Breeding of Special Aquatic Organisms, Huaiyin Normal University, Huai’an 223300, China; 4 Qingyuan Zhuoyue Aquatic Co., Ltd., Qingyuan 511500, China; 4376589@163.com

**Keywords:** *Channa argus*, northern snakehead, growth, body weight, GWAS, SNP array, candidate gene, *COL2A1*/collagen

## Abstract

Body weight is the principal economic trait in snakehead aquaculture, yet the genetic architecture of growth in the northern snakehead (*Channa argus*) remains largely unexplored. We performed a genome-wide association study (GWAS) for body weight in 205 cultured *C. argus* genotyped with a 50K SNP array. After quality control, 47,123 autosomal SNPs were tested under a univariate linear mixed model in GEMMA that accounted for genomic relatedness. One SNP on chromosome 7 (Chr07:1,329,732) exceeded the Bonferroni genome-wide threshold, and 14 SNPs reached a suggestive threshold, forming two candidate clusters on Chr07 and Chr24. Annotating candidate intervals (±100 kb) against the *C. argus* genome assembly yielded 93 candidate genes. Hypergeometric enrichment identified 44 Gene Ontology (GO) terms and 4 KEGG pathways, dominated by endolysosomal lumen, serine-type endopeptidase activity, collagen/extracellular-matrix catabolism, bone resorption, and the apoptosis, lysosome, phagocytosis and antigen-processing pathways. The single genome-wide significant SNP fell in an intron of a collagen alpha-1(II) chain gene (*COL2A1*/*zgc:113232*), consistent with the collagen/extracellular-matrix enrichment signal; other plausible growth-related candidates included *CTSS*, *S100A4*, *PYGM*, *DLAT*, *MSX2* and *ZNRF3*. The functional signal was largely driven by a tandem protease/cathepsin gene cluster on Chr07, indicating a regional rather than a genome-wide pathway effect. The lead SNP genotype was associated with a ~240 g (≈30%) difference in body weight, and this effect was reproduced in an independent cohort of 200 fish. These findings provide the first GWAS-based candidate loci for body weight in *C. argus* and a promising marker for future marker-assisted breeding, though validation to date is limited to a single additional cohort from the same broodstock source; broader multi-population testing will be needed before routine application.

## Simple Summary

The northern snakehead (*Channa argus*) is an economically important freshwater food fish in East Asia, and body weight is one of the most valuable traits for its aquaculture. Understanding which regions of the genome influence growth can help breeders select faster-growing fish more efficiently. In this study, we genotyped 205 northern snakeheads with a 50K single-nucleotide polymorphism (SNP) array and performed a genome-wide association study for body weight. We detected one genome-wide significant marker on chromosome 7 and several suggestive markers clustered on chromosomes 7 and 24. Genes located near these markers were enriched for functions related to lysosomal protein degradation, serine proteases, extracellular-matrix and collagen turnover, and apoptosis. These results provide an initial set of candidate genomic regions for growth in *C. argus* and a foundation for future marker-assisted selection, while also underscoring that larger populations will be needed to confirm the associations.

## 1. Introduction

Body weight, or growth, is among the most economically valuable target traits in aquaculture, directly determining the production cycle, harvest size and ultimate profitability [1]. Unlike qualitative traits controlled by a single major gene, body weight is a classic quantitative trait: its phenotype varies continuously and is governed by the additive effects of many loci of small to moderate effect, together with environmental conditions and management factors [1]. Genotype-by-environment interactions further complicate its genetic dissection, as the same genotype may grow differently under different culture environments or husbandry regimes [2]. Growth traits in fish generally show moderate heritability and are therefore amenable to selective improvement; however, because body weight can only be measured accurately near harvest and is simultaneously shaped by numerous loci of small effect and by environmental factors, conventional selection based solely on phenotypic records often progresses slowly and with limited accuracy, failing to fully capture this small-effect, widely distributed genetic variation [3]. In farmed fish, growth is the integrated outcome of multiple physiological processes—appetite, feed intake and conversion, muscle deposition, skeletal development and energy metabolism—so the genes underlying body-weight variation are expected to span multiple biological processes rather than a single pathway [1,3,4]. Dissecting this architecture is valuable on two fronts: it identifies the molecular pathways that limit growth and can serve as targets for management or selection, and it yields DNA markers that allow superior genotypes to be selected early and accurately, substantially shortening the breeding cycle relative to phenotypic selection [1,4,5]. For carnivorous, high-value species such as the snakehead—where feed accounts for the majority of production cost—even modest gains in growth or feed-conversion efficiency translate into substantial economic benefit [6], making the genetic dissection of body weight a priority for the emerging snakehead breeding sector.

Genome-wide association studies (GWAS) have become a standard tool for dissecting the genetic architecture of complex production traits in aquaculture species and have been applied to growth, disease resistance, sex determination and stress tolerance across a wide range of farmed fish and shellfish [1]. Growth-related loci and candidate genes have been mapped in tilapia [5,7,8,9], carps [10,11,12,13,14,15], salmonids [16,17,18,19,20], Pacific white shrimp [21], and molluscs such as oyster, scallop and abalone [22,23,24], as well as in many other teleosts including large yellow croaker [25,26], mandarin fish [27,28], estuarine tapertail anchovy [29], sea basses [30,31], Japanese flounder [32], channel catfish [3], blunt snout bream [33] and zig-zag eel [34]. For the closely related blotched snakehead (*Channa maculata*), a congener of *C. argus*, growth-associated SNPs and candidate genes [35], a high-density linkage map with growth and sex QTL [36], and a chromosome-level genome assembly [37] are already available. These advances have been driven by the development of medium- to high-density SNP arrays for many species [7,25,32], including snakeheads [38,39], and by chromosome-level reference assemblies that render association mapping feasible in non-model farmed fish. Linear mixed models that jointly correct for population structure and cryptic relatedness are now the standard analytical framework for such studies [40,41]. For *C. argus*, a recently developed 50K SNP array and a chromosome-anchored genome [37,39,42] together provide the resources required to interpret association signals at the gene level.

The northern snakehead (*Channa argus* Cantor, 1842) is a carnivorous freshwater teleost widely distributed across northern and southern China and intensively farmed throughout East Asia, prized for its rapid growth, high dietary protein content, and tolerance of hypoxia and high stocking densities. In recent years, the species and its hybrids have reached an annual production exceeding one million metric tons in China, commanding strong consumer demand and substantial commercial value [42,43]. Alongside this economic importance, genomic resources for *C. argus* have expanded rapidly. The mitochondrial genome was first assembled in 2013 [44], followed by a 615.3 Mb draft nuclear genome [45], a 630.39 Mb chromosome-level assembly [42], a 644.1 Mb assembly for the albino variant [46], and a high-quality 712.14 Mb reference genome [37]. These assemblies have in turn enabled the development of numerous trait-associated molecular markers, including markers for sex differentiation [47,48,49,50,51], growth [52], comparative sex-determination regions in *C. argus* and *C. maculata* [53], and yellow pigmentation [54], as well as the design of medium-density SNP arrays for both *C. argus* and the blotched snakehead *C. maculata* [38,39]. Despite this wealth of genomic data, genetic improvement of *C. argus* still relies largely on mass selection or conventional marker-assisted selection, approaches with limited power to improve polygenic, environmentally sensitive traits such as growth rate, flesh quality, and disease resistance, so that genetic progress remains slow. As market demand for premium, fast-growing snakehead continues to grow, there is a pressing need for more efficient molecular-breeding tools: cost-effective, high-throughput genotyping platforms such as SNP arrays can accelerate the discovery of trait-associated loci, enable genomic selection, and ultimately improve breeding efficiency for this commercially important species [1]. Body weight at harvest—the dominant breeding objective, as it governs feed-conversion economics and time to market—is a prime target for such marker-based approaches, yet its molecular basis in *C. argus* remains essentially unknown.

In this study, we used a custom 50K SNP array to genotype a cultured population of *C. argus* and conducted a GWAS for body weight under a linear mixed model correcting for cryptic relatedness. We then mapped association signals onto the annotated reference genome and performed Gene Ontology (GO) and Kyoto Encyclopedia of Genes and Genomes (KEGG) enrichment analyses on candidate genes. Our objectives were to (i) identify genomic regions and candidate genes associated with body weight, (ii) characterise the biological processes implicated by these candidates, and (iii) provide molecular markers and a methodological framework to support future marker-assisted selection in snakehead aquaculture.

## 2. Materials and Methods

### 2.1. Animals, Phenotyping and Ethics

Cultured northern snakeheads (*C. argus*) were obtained from a breeding population maintained at Qingyuan Zhuoyue Aquatic Co., Ltd. (Qingyuan, China), all originating from a single batch produced by family-based spawning. At harvest, 10 months post-hatch, a total of 205 males were sampled at random. Individual body weight (g) was recorded at harvest under standardised conditions and ranged from 591 to 1283 g (mean ± SD, 830.3 ± 138.4 g). A fin-clip was collected from each fish and stored in absolute ethanol at −20 °C until DNA extraction. All procedures involving animals were approved by the Institutional Animal Care and Use Committee of Pearl River Fisheries Research Institute, Chinese Academy of Fishery Sciences (LAEC-PRFRI-2026-06-20), and conducted in accordance with relevant national guidelines for the care and use of laboratory and farmed animals.

### 2.2. Genotyping and Quality Control

Genomic DNA was extracted from approximately 2 mg of fin tissue per individual using the DNeasy Blood & Tissue Kit (Qiagen, Valencia, CA, USA) following the manufacturer’s instructions, and DNA concentration and purity were assessed on a NanoDrop spectrophotometer (Thermo Fisher Scientific, Waltham, MA, USA). All samples were genotyped on a custom *C. argus* 50K SNP array according to the manufacturer’s protocol [39]. A targeted-capture (liquid-phase hybridization) sequencing-based platform (CAGT targeted-sequencing genotyping service, Kangpusen Biotechnology Co., Ltd., Beijing, China) was used rather than a fixed intensity-based chip; libraries were sequenced on a DNBSEQ-T7 platform (MGI/BGI, Shenzhen, China) with 150 bp paired-end reads, and genotypes were called from the aligned sequencing reads using the GATK v4.6.2.0 HaplotypeCaller/GenotypeGVCFs joint-genotyping workflow. Variant calls were generated against the chromosome-level *C. argus* reference assembly (GCF_033026475.1). Quality control was performed in PLINK v1.9 [55] with the following filters: SNP call rate ≥ 0.90 (–geno 0.1), minor-allele frequency ≥ 0.05 (–maf 0.05), and restriction to the 24 assembled autosomes. After QC, 49,755 SNPs and 205 individuals were retained; 47,123 SNPs were informative for the association analysis. All 205 individuals passed sample-level sequencing QC with a narrow, uniformly high-quality distribution (clean/effective-read ratio 99.47–99.77%, Q20 99.27–99.59%, Q30 97.45–98.51%, mapping rate 99.98–99.99%, properly paired rate 82.24–96.33%, PCR duplication rate 1.85–4.18%; per-sample ranges), and no individual required exclusion on sequencing-quality grounds.

### 2.3. Genome-Wide Association Analysis

Association testing was performed with GEMMA v0.98.5 [41] using a univariate linear mixed model (LMM):

**y = Wα + xβ + u + ε**, with **u ~ N(0, λτ^−1^K)** and **ε ~ N(0, τ^−1^I),**

where **y** is the vector of body-weight phenotypes, **W** is a matrix of covariates (intercept), **x** is the marker genotype, **β** is its allelic effect, **u** is the vector of random polygenic effects, **K** is the centred genomic relatedness matrix estimated from all QC-passed SNPs, and **ε** is the residual. SNP effects were tested with the Wald test (-lmm 1). Genomic inflation was assessed via the genomic control factor λ_GC computed from the median of the observed χ^2^ statistics.

A conservative Bonferroni-corrected genome-wide significance threshold was set at 0.05/47,123 = 1.06 × 10^−6^, and a suggestive threshold at *p* < 1 × 10^−4^. Manhattan and quantile–quantile plots were produced using CMplot v4.5.1 in R v4.6.1 [56].

### 2.4. SNP Annotation and Candidate-Gene Identification

Significant and suggestive markers were positionally annotated against the *C. argus* gene models using BEDTools v2.30.0 [57]. Each was classified as exonic (CDS or UTR), intronic, or intergenic; genic markers were assigned to the gene containing them, and intergenic markers to the nearest gene together with the distance to it. Linkage disequilibrium was characterised using PLINK v1.9: pairwise r^2^ was computed for all autosomal marker pairs ≤ 300 kb apart, averaged in 5 kb bins and fitted by exponential decay, and the distance at which mean r^2^ reached the background level of 0.1 was taken as the extent of genome-wide LD. On this basis, all protein-coding genes within ± 100 kb of a suggestive marker were defined as the candidate gene set, capturing *cis*-regulatory candidates while accommodating local LD. Pairwise r^2^ was also computed among suggestive markers within each focal interval to test whether they represented independent signals.

### 2.5. Functional Annotation and Enrichment Analysis

Functional annotation of all 24,608 predicted proteins was performed with eggNOG-mapper v2.1.13 [58] (DIAMOND mode [59]; eggNOG 5.0 orthology database [60]), yielding GO [61] and KEGG [62] pathway assignments. GO annotations were propagated to all ancestor terms using the go-basic ontology. Over-representation of GO terms and KEGG pathways in the candidate gene set was tested by the hypergeometric test (one-sided), using all functionally annotated genes as the statistical background. *p*-values were corrected for multiple testing by the Benjamini–Hochberg false-discovery-rate (FDR) procedure [63], and terms with FDR < 0.05 supported by ≥2 candidate genes were considered significantly enriched. Enrichment results were visualised as bubble and bar plots in Python v3.11 using matplotlib v3.11.1.

### 2.6. Validation of the Lead SNP in an Independent Population

To validate the lead association at Chr07:1,329,732, an independent cohort of 200 cultured *C. argus* from Qingyuan Zhuoyue Aquatic Co., Ltd. was genotyped at this locus using Sanger sequencing, and individual body weight was recorded under the same protocol as the discovery panel. Body weight was compared between the T/A and T/T genotype groups (100 individuals each) with Welch’s two-sample *t*-test.

## 3. Results

### 3.1. Sequencing, Genotyping and Data Quality

Array genotyping yielded high-quality data across all individuals (Table 1 and Figure S1). On average, each sample produced 2.9 Gb of clean sequence (19.9 M clean reads), with an effective ratio of 99.61%, Q30 of 98.12% and a GC content of 40.59%. Reads mapped to the *C. argus* reference at a rate of 99.99% (89.75% properly paired) with a low duplication rate (2.89%). Of the 49,988 target sites represented on the 50K array, 99.95% were covered at ≥1× and 99.86% at ≥20× per individual, indicating near-complete and uniform interrogation of the designed markers. After quality control (call rate ≥ 0.90, MAF ≥ 0.05, autosomes only), 49,755 SNPs were retained, of which 47,123 were informative for association testing.

**Figure 1:**
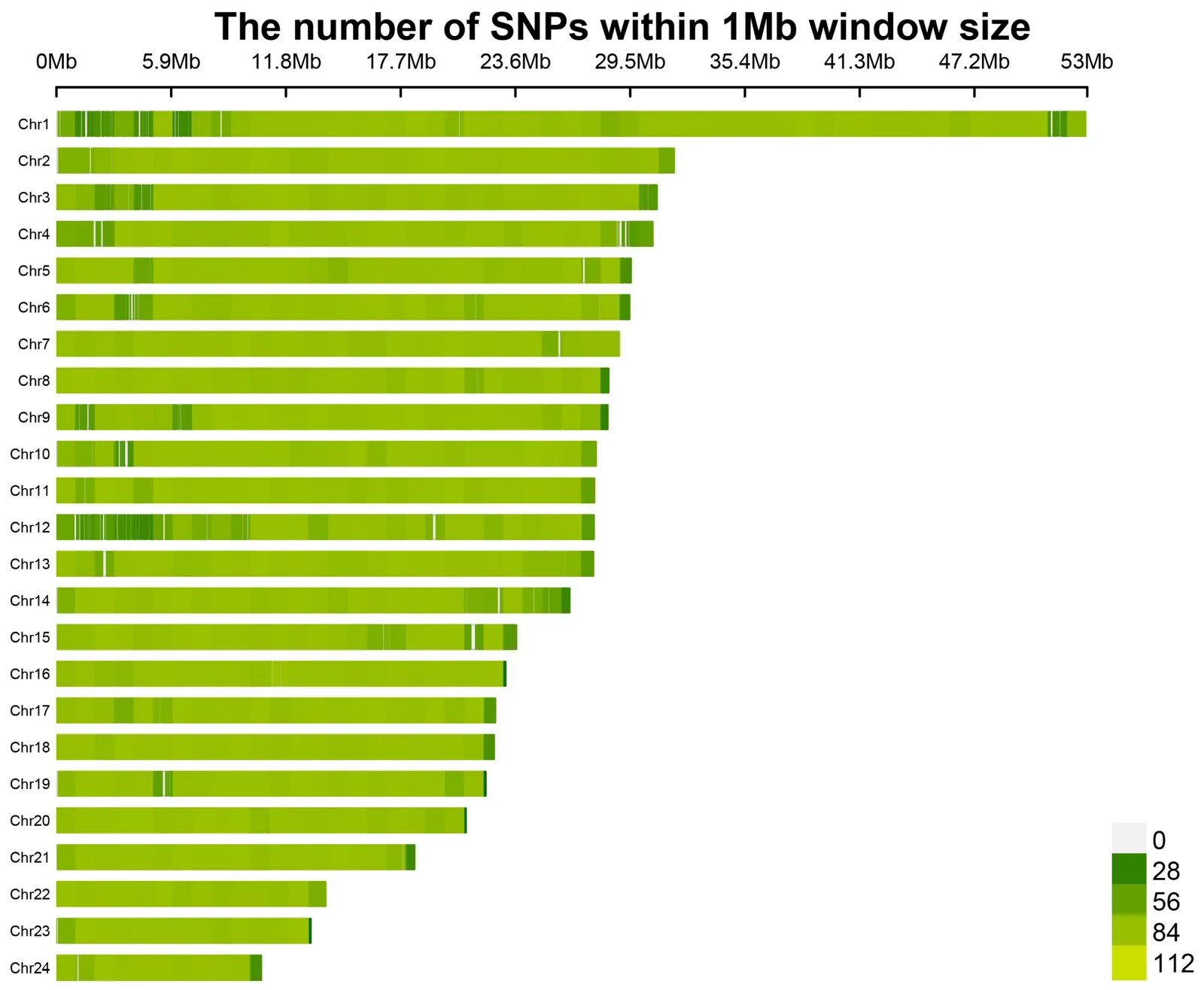
Genome-wide marker density of the 50K array, drawn with CMplot as the number of SNPs within each 1 Mb window along the 24 chromosomes.

The autosomal SNPs retained after QC were distributed across all 24 chromosomes, with a median density of ~76 SNPs per Mb and no large coverage gaps (Figure 1), confirming a genome-wide marker representation suitable for association mapping.

### 3.2. Phenotypic Variation and Model Diagnostics

**Figure 2:**
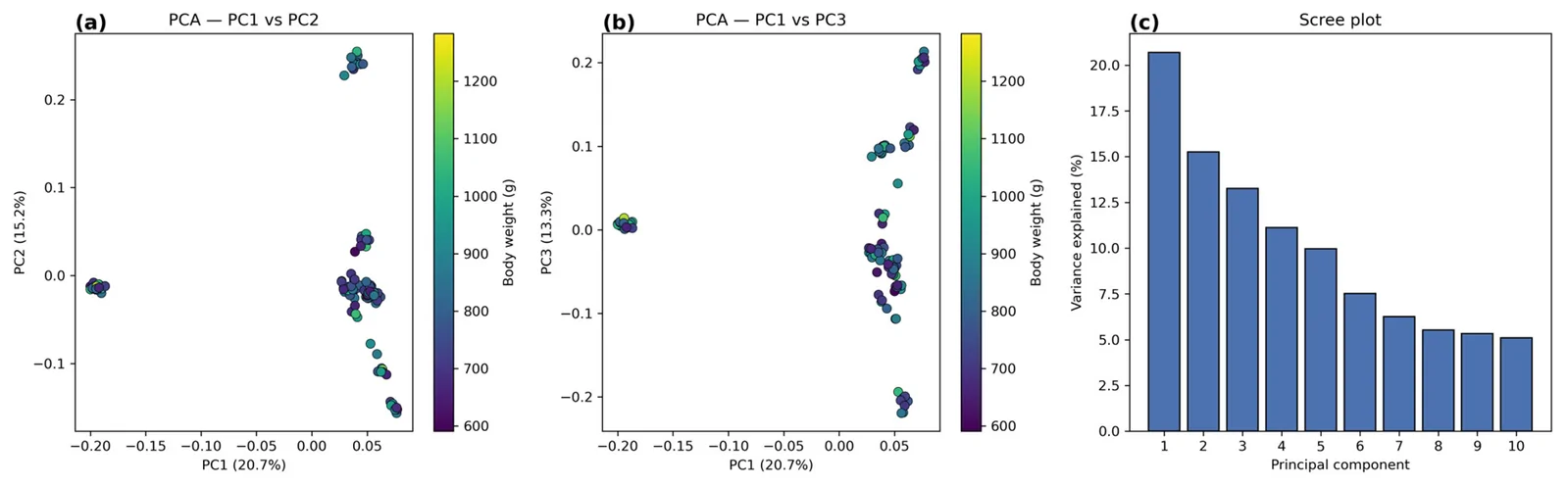
Principal component analysis of the genotyped population, coloured by body weight: (**a**) PC1 vs. PC2; (**b**) PC1 vs. PC3; (**c**) scree plot of variance explained.

Body weight in the genotyped *C. argus* showed substantial variation (591–1283 g; mean 830.3 ± 138.4 g; Figure S1a), providing adequate phenotypic spread for association analysis. Principal component analysis of 24,034 LD-pruned SNPs revealed no major population stratification, and body weight was not associated with any of the leading principal components (PC1 = 20.7%, PC2 = 15.3%, PC3 = 13.3% of variance); a small number of individuals separated along PC1, most likely reflecting familial relatedness (Figure 2). The LMM was well calibrated, with a genomic inflation factor λ_GC = 1.083, indicating only minor residual stratification after correction for the genomic relatedness matrix.

### 3.3. Genome-Wide Association Signals

**Figure 3:**
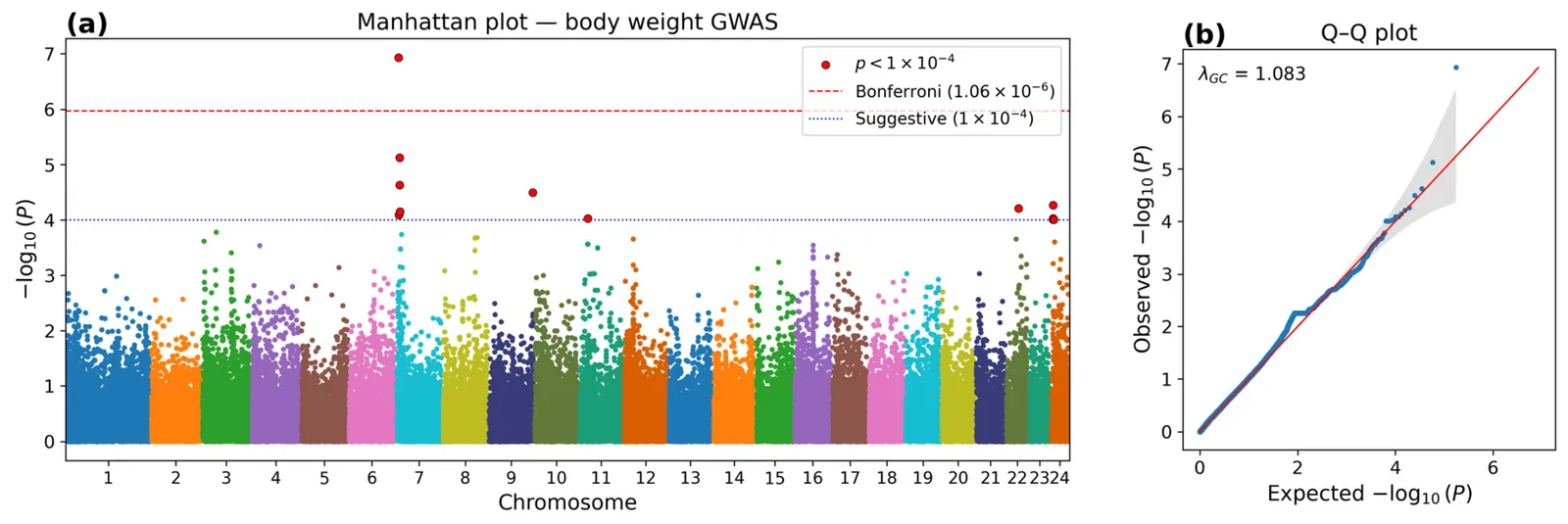
(**a**) Manhattan plot and (**b**) Q–Q plot (λ_GC = 1.083; red 1:1 expectation line and grey 95% confidence band) for the body-weight GWAS; in (**a**), red points mark SNPs with *p* < 1 × 10^−4^, the dashed red line marks the Bonferroni threshold, and the dotted blue line marks the suggestive threshold.

**Figure 4:**
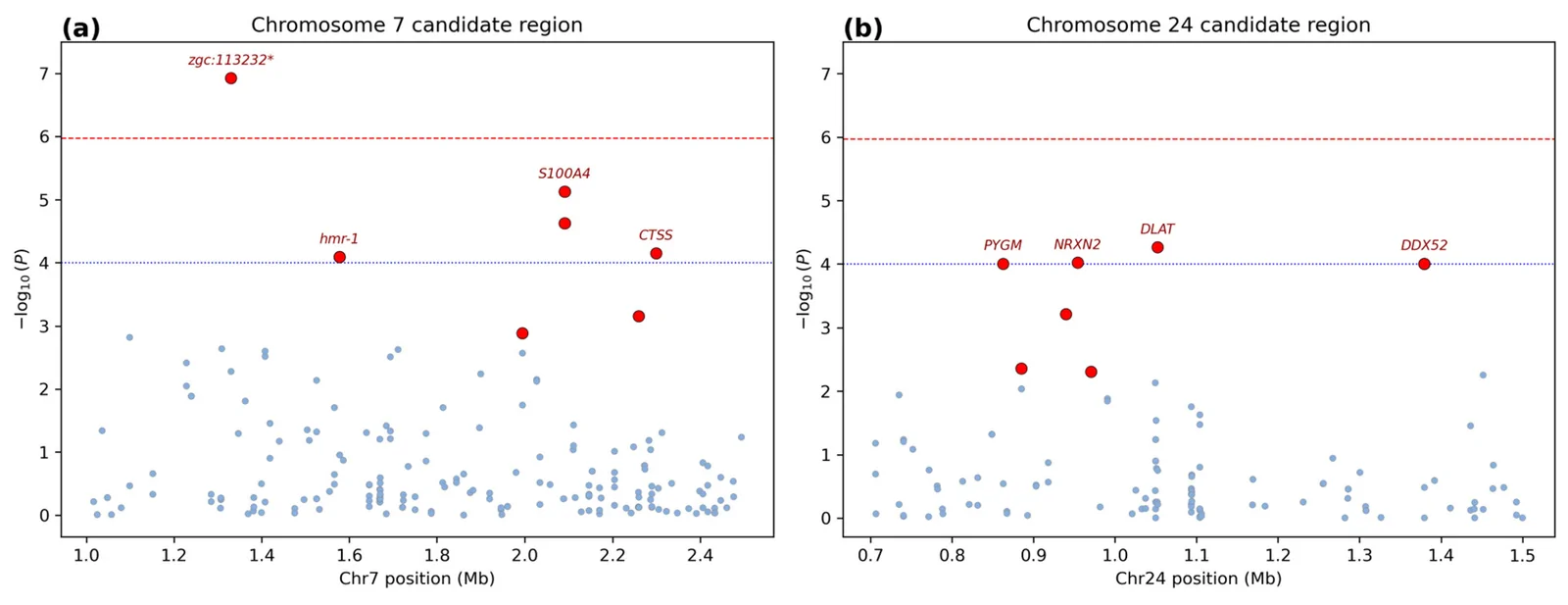
Regional association plots for the candidate intervals on (**a**) chromosome 7 and (**b**) chromosome 24. Red points indicate the lead SNPs, whereas light-blue points indicate the remaining SNPs. The dashed red and dash-dotted blue horizontal lines denote the Bonferroni genome-wide significance threshold and the suggestive threshold, respectively. The asterisk marks the genome-wide significant lead SNP, and italic labels indicate the nearest annotated genes.

Of the 47,123 SNPs analysed, one marker exceeded the Bonferroni genome-wide significance threshold: Chr07:1,329,732 (*p*_wald = 1.18 × 10^−7^), located within an intron of *Chr07.g07382*, which encodes collagen alpha-1(II) chain (*COL2A1*; *zgc:113232*). A further 13 SNPs reached the suggestive threshold (*p* < 1 × 10^−4^), and these associations were not randomly distributed but formed two candidate clusters, on chromosome 7 (1.30–2.30 Mb) and chromosome 24 (0.86–1.38 Mb), together with single suggestive signals on chromosomes 9, 11 and 22 (Table 2 and Figure 3). Regional association plots showed multiple sub-threshold markers within both intervals (Figure 4), a pattern more readily explained by quantitative trait loci tagged through linkage disequilibrium than by isolated false positives.

Mean r^2^ declined from 0.45 between adjacent markers to the background level (r^2^ = 0.1) at ~68 kb and plateaued thereafter at the fitted asymptote of 0.086, with binned estimates stable at background out to 300 kb (Figure S2). Since LD had reached background well within 100 kb, the ± 100 kb window used to define candidate genes (Section 2.4) is conservative genome-wide, although local LD proved more extensive. On chromosome 7, the lead variant was in moderate LD with the nearest suggestive marker (r^2^ = 0.43 at ~248 kb) and in weaker LD (r^2^ = 0.27–0.40) with markers up to ~760 kb, including those near *S100A4* (Table 2). On chromosome 24, *PYGM*, *NRXN2*, *DLAT* and *DDX52* formed a single high-LD block (r^2^ = 0.49–1.00 across ~517 kb), indicating one haplotype rather than four independent signals.

### 3.4. Allelic Effect of the Lead SNP on Body Weight and Independent Validation

To visualise the effect of the genome-wide significant marker, the 205 individuals were grouped by their genotype at Chr07:1,329,732 (an intronic SNP in *COL2A1*, alleles T/A). The variant was common: 145 fish were heterozygous T/A; 60 were homozygous T/T. Body weight increased markedly with the number of T alleles: T/A fish averaged 808.2 ± 119.7 g, whereas T/T homozygotes were substantially heavier (T/T, 1047.2 ± 135.9 g)—a difference of ~240 g (≈30%) between T/A and T/T fish (Welch’s *t*-test, *t* = −5.09, *p* = 6.0 × 10^−4^). This per-genotype pattern is fully concordant with the GEMMA mixed-model result (*p*_wald = 1.18 × 10^−7^) and indicates that the T allele is associated with higher body weight, although the small number of carriers in the discovery panel warrants cautious interpretation.

**Figure 5:**
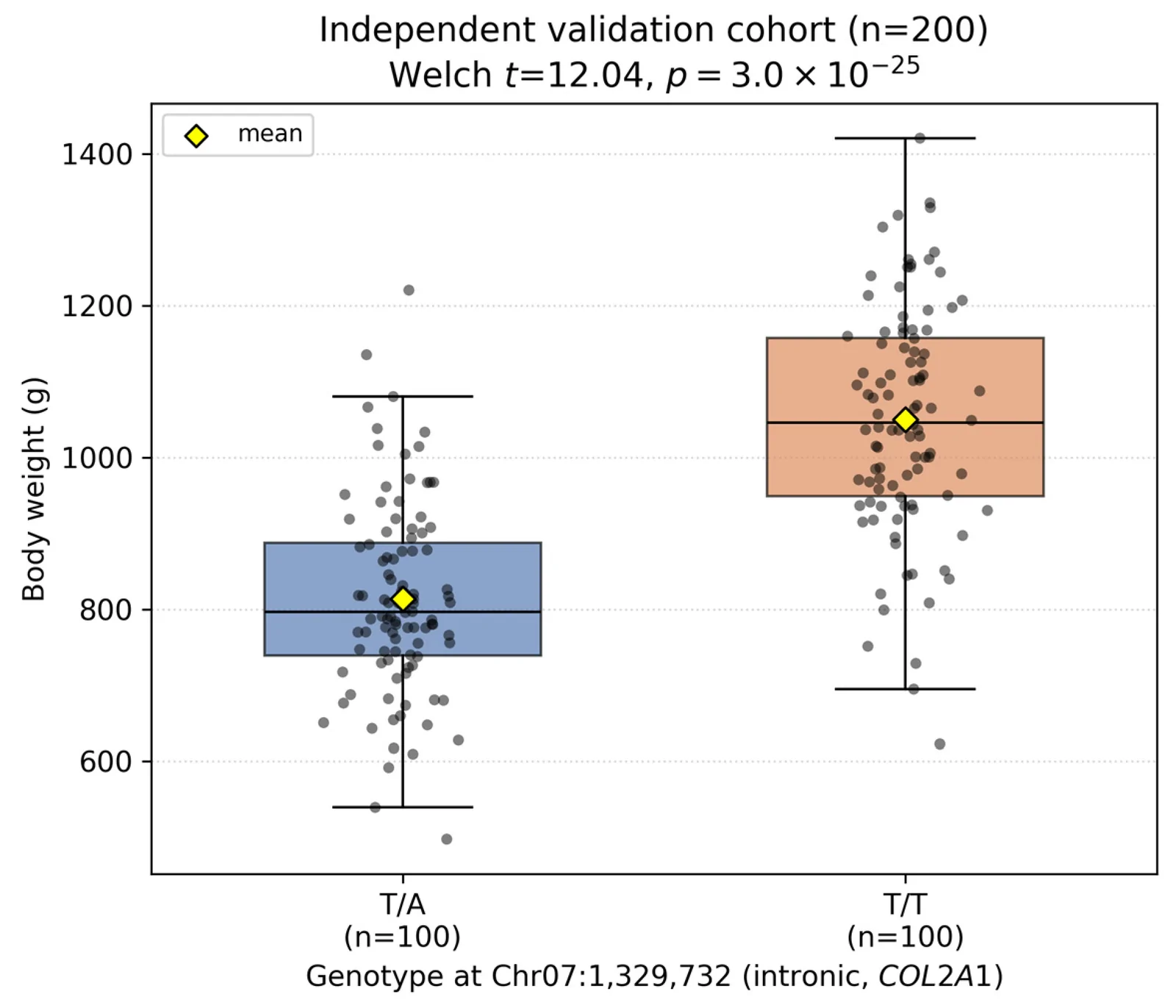
Body weight by genotype at the lead SNP Chr07:1,329,732 (intronic, *COL2A1*) in an independent validation cohort (*n* = 200; 100 T/A and 100 T/T). Boxes show the median and interquartile range, black points individual fish, and yellow diamonds the group means; group sizes are given on the x-axis. T/T carriers are ~237 g (≈29%) heavier than T/A heterozygotes (Welch’s *t* = 12.04, *p* = 3.0 × 10^−25^).

To test the robustness of this association, we genotyped the Chr07:1,329,732 variant in an independent validation cohort of 200 *C. argus* (100 T/A and 100 T/T) and compared body weight between genotypes. The effect was clearly reproduced: T/A fish averaged 813.4 ± 126.9 g versus 1050.1 ± 150.2 g in T/T carriers—a difference of 236.7 g (29.1%), closely matching the ~240 g (≈30%) difference observed in the discovery cohort and highly significant in this balanced sample (Welch’s *t*-test, *t* = 12.04, *p* = 3.0 × 10^−25^; Figure 5). This independent replication provides additional support regarding the evidence that the T allele at the *COL2A1* lead SNP is associated with higher body weight in *C. argus*.

### 3.5. Functional Annotation of Candidate Genes

eggNOG-mapper assigned functional annotations to 22,436 of the 24,608 predicted *C. argus* proteins (91.2%), including 15,327 genes with GO terms and 9275 with KEGG pathway memberships. The 93 candidate genes within ±100 kb of suggestive SNPs fell into several functionally coherent groups (Table 1). The first comprises genes of skeletal, cartilage and extracellular-matrix biology: the lead gene *COL2A1* (collagen alpha-1(II) chain), annotated to ECM–receptor interaction, focal adhesion, and protein digestion and absorption; the skeletal transcription factor *MSX2* (muscle-segment homeobox 2); and the cadherin *hmr-1*, a Ca^2+^-dependent cell-adhesion protein implicated in cell growth and morphogenesis. A second group is involved in protein catabolism and tissue remodelling, dominated by the cathepsin *CTSS* and its neighbouring protease paralogues on Chr07, together with the calcium-binding protein *S100A4* (annotated to epithelial-to-mesenchymal transition and actin binding). A third group acts in energy and carbohydrate metabolism: muscle glycogen phosphorylase *PYGM*, which is rate-limiting for glycogenolysis and is itself annotated to “response to hypoxia” and the insulin/glucagon signalling pathways, and *DLAT*, the E2 subunit of the pyruvate-dehydrogenase complex linking glycolysis to the TCA cycle. The remaining candidates include the Wnt-pathway regulator *ZNRF3* (a frizzled-binding ubiquitin ligase), the synaptic adhesion molecule *NRXN2* (neurexin 2a), the RNA helicase *DDX52*, and the vesicle-transport protein *CCDC93*.

### 3.6. GO and KEGG Enrichment

**Figure 6:**
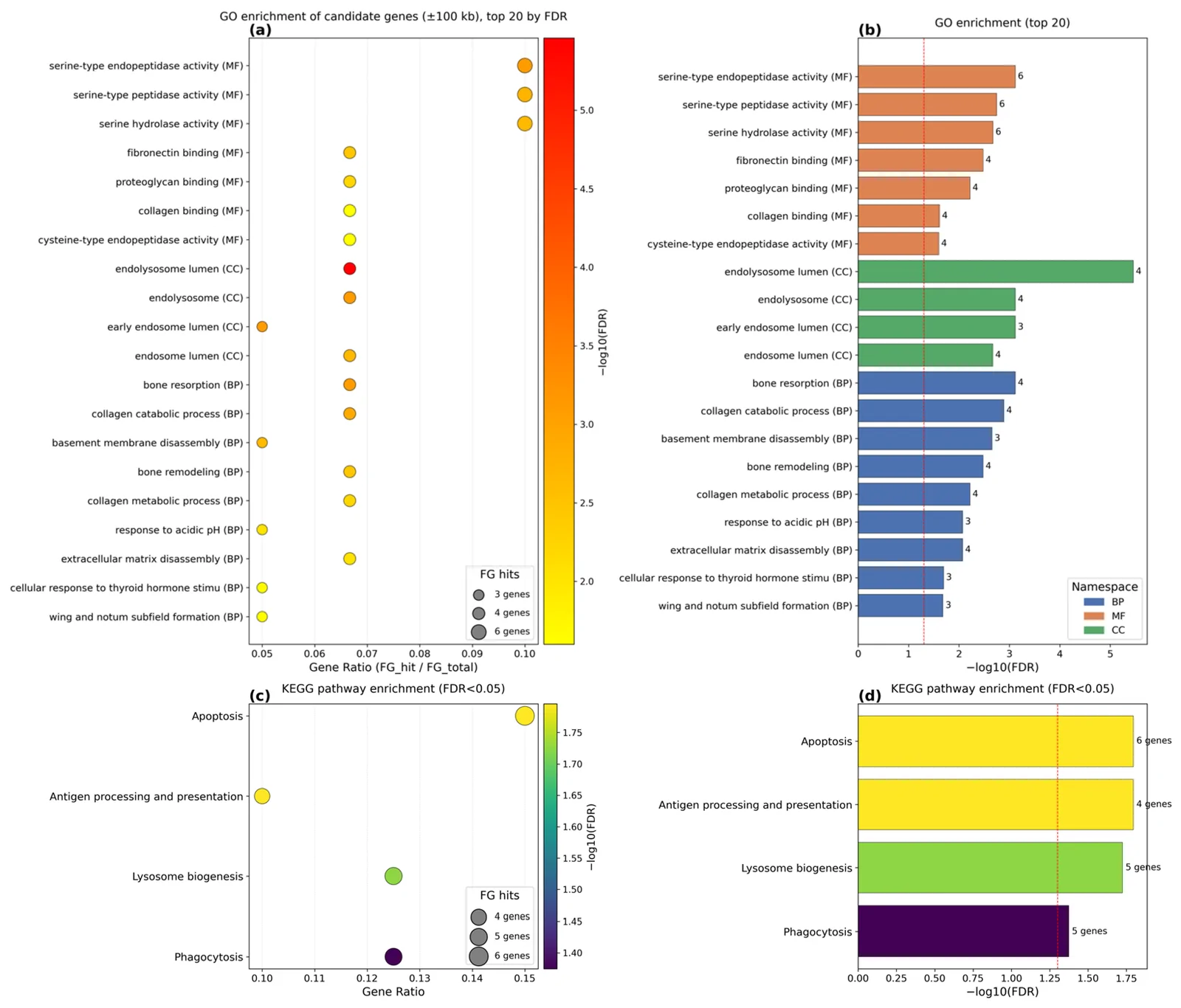
**Functional enrichment of body-weight candidate genes.** (**a**) GO bubble plot and (**b**) GO bar plot of the top 20 enriched GO terms; (**c**) KEGG bubble plot and (**d**) KEGG bar plot. In the bubble plots, the x-axis represents the gene ratio, bubble size indicates the number of candidate genes, and bubble color represents −log_10_(FDR). In panel (**b**), bar colors indicate the GO namespaces: biological process (BP), molecular function (MF), and cellular component (CC). In panel (**d**), bar color represents −log_10_(FDR). Numbers at the ends of the bars indicate the numbers of candidate genes, and the red dashed line denotes the FDR = 0.05 threshold.

Hypergeometric testing identified 44 GO terms (FDR < 0.05) over-represented among candidate genes (Figure 6a,b). The strongest signals were in the cellular-component category—endolysosome lumen (FDR = 3.5 × 10^−6^), early endosome lumen (FDR = 7.6 × 10^−4^), endolysosome and endosome lumen—pointing to the lumen of the endolysosomal degradation system. The molecular-function category was dominated by peptidase activities, including serine-type endopeptidase activity (FDR = 7.6 × 10^−4^), serine-type peptidase activity, serine hydrolase activity, cysteine-type endopeptidase activity (FDR = 0.025) and broad endopeptidase activity, alongside matrix-binding terms—fibronectin binding (FDR = 3.3 × 10^−3^), proteoglycan binding, collagen binding and laminin binding. Enriched biological processes centred on skeletal turnover and protein catabolism: bone resorption (FDR = 3.5 × 10^−6^), collagen catabolic process (FDR = 1.3 × 10^−3^), bone remodelling (FDR = 3.3 × 10^−3^), collagen metabolic process, basement membrane disassembly, extracellular matrix disassembly, tissue remodelling and cranial suture morphogenesis. Notably, response to thyroid hormone and response to acidic pH were also enriched, as both processes have direct relevance to teleost growth and lysosomal/cathepsin activity. Four KEGG pathways were significantly enriched (FDR < 0.05): apoptosis (6 genes; FDR = 0.016), antigen processing and presentation (4 genes; FDR = 0.016), lysosome (5 genes; FDR = 0.019) and phagocytosis (5 genes; FDR = 0.042); the PI3K-Akt signalling pathway (7 genes, including *COL2A1*) was borderline (FDR = 0.052) (Figure 6c,d). Consistent with a regional rather than a genome-wide effect, the genes driving the enriched protease/lysosome terms were largely a contiguous block of paralogues on Chr07 (Chr07.g07421–g07424 and neighbours), a point examined further in the Discussion section.

## 4. Discussion

This study presents, to our knowledge, the first genome-wide association analysis of body weight in the northern snakehead and identifies two candidate genomic regions— on chromosomes 7 and 24—together with a set of biologically plausible candidate genes. The convergence of multiple suggestive markers within narrow intervals, the acceptable genomic inflation (λ_GC = 1.083), and the coherent functional enrichment of the underlying genes collectively suggest that these regions harbour real, if modest, determinants of growth.

Several candidate genes have well-documented roles in growth and energy metabolism. *PYGM* (muscle glycogen phosphorylase) is rate-limiting for glycogenolysis and directly influences muscle energy supply and, ultimately, muscle mass. *DLAT*, the E2 component of the pyruvate-dehydrogenase complex, links glycolysis to the citric-acid cycle and oxidative ATP production; energy-metabolism genes of this kind have repeatedly emerged as growth-associated candidates in teleost GWAS [4,15,27]. *S100A4* and the cathepsin *CTSS* participate in tissue remodelling and extracellular-matrix turnover, processes intimately coupled to muscle hypertrophy and somatic growth in fish [3,4], while *MSX2* and *ZNRF3* (a negative regulator of Wnt/β-catenin signalling) are involved in skeletal patterning and developmental growth control. Most notably, the single genome-wide significant locus (Chr07:1,329,732) lies within an intron of *Chr07.g07382*, which encodes collagen alpha-1(II) chain (*COL2A1*; *zgc:113232*; best mRNA hit to the anabantoid *Anabas testudineus*). COL2A1 is the principal fibrillar collagen of cartilage and is essential for chondrogenesis, skeletal growth and endochondral ossification. In teleosts, *col2a1a* is expressed in craniofacial cartilage, the notochord, and fins and is required for normal notochord and vertebral development [64,65], and reduced *col2a1* expression is associated with lower-jaw skeletal deformity in farmed Atlantic salmon [66]; collectively, these data make *COL2A1* a compelling functional candidate for body-weight and skeletal variation in fish. Critically, this assignment is internally consistent with the enrichment results, in which collagen catabolic process, extracellular-matrix disassembly and bone resorption/remodelling were among the most over-represented terms—pointing to collagen and skeletal-matrix turnover as a recurring theme at this locus. Because the lead variant is intronic (non-coding), it most plausibly acts by modulating *COL2A1* expression or through linkage with nearby regulatory variants, rather than by altering the protein sequence.

The second candidate region, on chromosome 24 (≈ 0.86–1.38 Mb), is biologically distinct from the Chr07 collagen/protease block and is metabolically themed. It harbours *PYGM*, *DLAT*, *NRXN2* and *DDX52* within a narrow interval. The co-localisation of two core energy-metabolism enzymes—*PYGM*, which mobilises muscle glycogen, and *DLAT*, which channels pyruvate into oxidative phosphorylation—is notable because muscle energy provisioning is a direct determinant of growth rate and feed-conversion efficiency in carnivorous teleosts [6]. *PYGM* is itself annotated to “response to hypoxia”, a trait of particular relevance to the hypoxia-tolerant snakehead, raising the possibility that the same locus contributes to both growth and hypoxia performance, as has been reported for growth/hypoxia QTL co-localisation in the Chinese longsnout catfish and in blunt snout bream [4,33,67]. The Chr24 signal, although only suggestive here, therefore warrants targeted fine-mapping.

Placed in a comparative context, the candidate genes identified here echo recurrent themes from growth GWAS across farmed teleosts worldwide, not only in Chinese aquaculture species. Energy-metabolism genes have been repeatedly nominated in bighead carp, mandarin fish, and Chinese longsnout catfish [4,15,27], similarly in Nile tilapia from Chile and international WorldFish/Roslin Institute collaborations [5,9], and in rainbow trout from the United States [19,20]; extracellular-matrix, protease, and tissue-remodelling genes feature prominently in channel catfish and longsnout catfish growth studies [3,4], as well as in a genome-wide association study of Eastern oyster growth from Canada [22]; skeletal/collagen genes recur in salmonid growth and deformity work [18,66]. The convergence of our snakehead candidates on these same functional axes—muscle energy metabolism, ECM/collagen turnover and lysosomal proteolysis—rather than on a single canonical growth-hormone/IGF axis is consistent with the highly polygenic and species-contingent architecture now documented for body weight in fish, in which different populations and species highlight different members of a shared set of growth-related pathways.

The functional enrichment results paint a consistent picture centred on lysosomal and proteolytic protein turnover, extracellular-matrix/collagen catabolism, and apoptosis—processes that govern the balance between tissue synthesis and degradation and thereby net growth. Lysosomal and autophagic protein degradation in particular is a recognised regulator of muscle mass and growth efficiency in teleosts [3,4]. However, an important methodological caveat applies: much of the GO/KEGG signal is contributed by a tandem array of cathepsin/serine-protease genes located within the single Chr07 candidate interval. Because physically clustered, functionally related genes are co-selected as candidates, standard over-representation tests can inflate the apparent significance of the corresponding terms. The enrichment should therefore be interpreted as identifying a candidate region rich in protease/lysosomal genes, rather than as evidence of an independent, genome-wide pathway effect. Gene-set tests that account for gene clustering and LD (e.g., MAGMA-style or eigen-decomposition approaches) would be valuable in future work.

From a breeding standpoint, the validated *COL2A1* marker is a promising, though still preliminary, candidate for marker-assisted selection. The favourable T allele was associated with a ~29–30% increase in body weight in both the discovery and the 200-fish validation cohorts, an effect large enough to be exploited by marker-assisted selection even before the polygenic background is characterised [1,4]. Importantly, the T allele is currently rare in the studied population, so its frequency—and hence the population mean body weight—could in principle be raised rapidly through marker-assisted matings, provided the association is confirmed across additional broodstocks and the allele carries no antagonistic effects on fitness, skeletal integrity or product quality (a relevant concern given the gene’s role in cartilage and vertebral development) [66]. A KASP or similar low-cost assay for this single SNP would be straightforward to deploy in a commercial hatchery setting, as demonstrated for growth markers in blunt snout bream [33].

The principal limitation of this study is the modest sample size (*n* = 205), which constrains statistical power and restricts detection at genome-wide significance to loci of relatively large effect. Small discovery panels are also prone to inflated effect-size estimates (the Beavis effect) and to missing the many small-effect loci expected for a polygenic trait such as growth. Consequently, the loci reported here should be regarded as candidate associations requiring replication. Future studies should (i) expand the genotyped population and incorporate additional growth traits (body length, condition factor, growth rate); (ii) validate the Chr07 and Chr24 loci in independent cohorts and via fine-mapping and LD analysis; and (iii) evaluate the predictive value of the candidate markers for genomic or marker-assisted selection.

## 5. Conclusions

This study provides the first genome-wide evidence for the genetic architecture of body weight in the northern snakehead, identifying a replicated, genome-wide significant locus and a biologically coherent set of candidate genes for this economically important trait. Using a 50K SNP array and a linear-mixed-model GWAS in 205 northern snakeheads, we identified one genome-wide significant locus on chromosome 7—an intronic variant in the collagen alpha-1(II) chain gene (*COL2A1*/*zgc:113232*)—and a second suggestive candidate region on chromosome 24 associated with body weight, and we nominated several further plausible growth-related candidate genes (*CTSS*, *S100A4*, *PYGM*, *DLAT*, *MSX2*, *ZNRF3*). Functional enrichment implicated collagen/extracellular-matrix turnover, lysosomal/proteolytic protein degradation, and apoptosis, consistent with the collagen identity of the lead gene, although the signal was largely driven by a Chr07 protease/collagen gene cluster and should be interpreted as region-level rather than genome-wide pathway evidence. The genotype–phenotype effect of the lead *COL2A1* SNP was confirmed in an independent cohort of 200 fish, in which T/T carriers were ~240 g (≈30%) heavier than T/A heterozygotes (*p* = 3.0 × 10^−25^). These results provide the first GWAS-based candidate loci, an independently validated molecular marker for growth in *C. argus*, and a framework for future marker-assisted breeding, while broader multi-population validation will further refine their utility.

## Figures and Tables

**Table 1:** Summary of sequencing, mapping, target-coverage and variant statistics for the 50K array genotyping of *C. argus* (chip_seq_summary.tsv).

Metric	Value (Mean ± SD or Range)
Clean bases per sample (Gb)	2.9 (1.6–4.0)
Clean reads per sample (M)	19.9 (10.9–27.4)
Effective ratio (%)	99.61 ± 0.05
Q20/Q30 (%)	99.46 ± 0.07/98.12 ± 0.22
GC content (%)	40.59 ± 0.12
Mapping rate (%)	99.99 ± 0.00
Properly mapped (%)	89.75 ± 2.26
Duplication rate (%)	2.89 ± 0.39
Target sites on array	49,988
Target sites ≥1×/≥10×/≥20× (%)	99.95/99.89/99.86
Variants after QC	49,755
Autosomal SNPs analysed (GWAS)	47,123

**Table 2:** Significant and suggestive SNPs associated with body weight in *C. argus* and their gene annotations.

Chr	Position	*p*_wald	Class	Gene	Region	Nearest-Gene Symbol	Description
7	1,329,732	1.18 × 10^−7^	Bonferroni	***Chr07.g07382***	intronic	*COL2A1* (*zgc:113232*)	collagen alpha-1(II) chain (best mRNA hit *Anabas testudineus*)
7	2,090,978	7.51 × 10^−6^	suggestive	***Chr07.g07413***	intergenic (±11.3 kb)	*S100A4*	S-100 calcium-binding protein
7	2,090,577	2.36 × 10^−5^	candidate	***Chr07.g07412***	intergenic (±11.3 kb)	—	uncharacterised
9	28,017,478	3.20 × 10^−5^	candidate	***Chr09.g10369***	intronic	*CCDC93*	coiled-coil domain containing 93
24	1,051,987	5.47 × 10^−5^	candidate	***Chr24.g23430***	intronic	*DLAT*	pyruvate dehydrogenase complex E2
22	7,762,258	6.17 × 10^−5^	candidate	***Chr22.g22629***	intergenic (±18.1 kb)	*MSX2*	muscle-segment homeobox 2
7	2,298,826	7.15 × 10^−5^	candidate	***Chr07.g07424***	intronic	*CTSS*	cathepsin S
7	1,577,548/549	8.14 × 10^−5^	candidate	***Chr07.g07397***	intronic	*hmr-1*/cadherin	Ca^2+^-dependent cell adhesion
11	5,157,585	9.46 × 10^−5^	candidate	***Chr11.g11491***	intergenic (±3.8 kb)	*ZNRF3*	zinc/ring-finger 3 (Wnt regulator)
24	953,970	9.51 × 10^−5^	candidate	***Chr24.g23427***	intronic	*NRXN2*	neurexin 2a
24	862,373	9.92 × 10^−5^	candidate	***Chr24.g23426***	intronic	*PYGM*	muscle glycogen phosphorylase
24	1,379,438/451	9.92 × 10^−5^	candidate	***Chr24.g23451***	intergenic (±57–70 bp)	*DDX52*	DEAD-box helicase 52

## Data Availability

The genotype and phenotype datasets generated and analysed in this study are available from the corresponding author on reasonable request.

## Supplementary Materials

The following supporting information can be downloaded at: https://www.mdpi.com/article/10.3390/ani16162471/s1, Figure S1: Phenotype and data-quality overview: (a) body-weight distribution; (b) per-sample Q20/Q30/GC; (c) mapping rates; (d) target-site (50K) coverage (Fig1_pheno_QC.png). Figure S2: Genome-wide linkage-disequilibrium (LD) decay in the discovery cohort (*n* = 205): mean pairwise r^2^ (PLINK v1.9) across all autosomal SNP pairs within 300 kb, binned in 5 kb windows, with a fitted exponential decay curve (r^2^ = 0.366·e^(−0.048·d[kb]) + 0.086). The dashed line marks the conventional r^2^ = 0.1 background-LD threshold, reached at ~70–100 kb.
