# Supplementary material for: A Genome-Wide Association Study Identifies Candidate SNPs and Genes Associated with Body Weight in the Northern Snakehead (Channa argus) Using a 50K SNP Array
Source: Animals (Basel). 2026 Aug 8;16(16):2471. doi: 10.3390/ani16162471 (PMC13508929; doi:10.3390/ani16162471)
Supplement: Supplementary file 1 [file animals-16-02471-s001.zip › FigureS1.pdf]

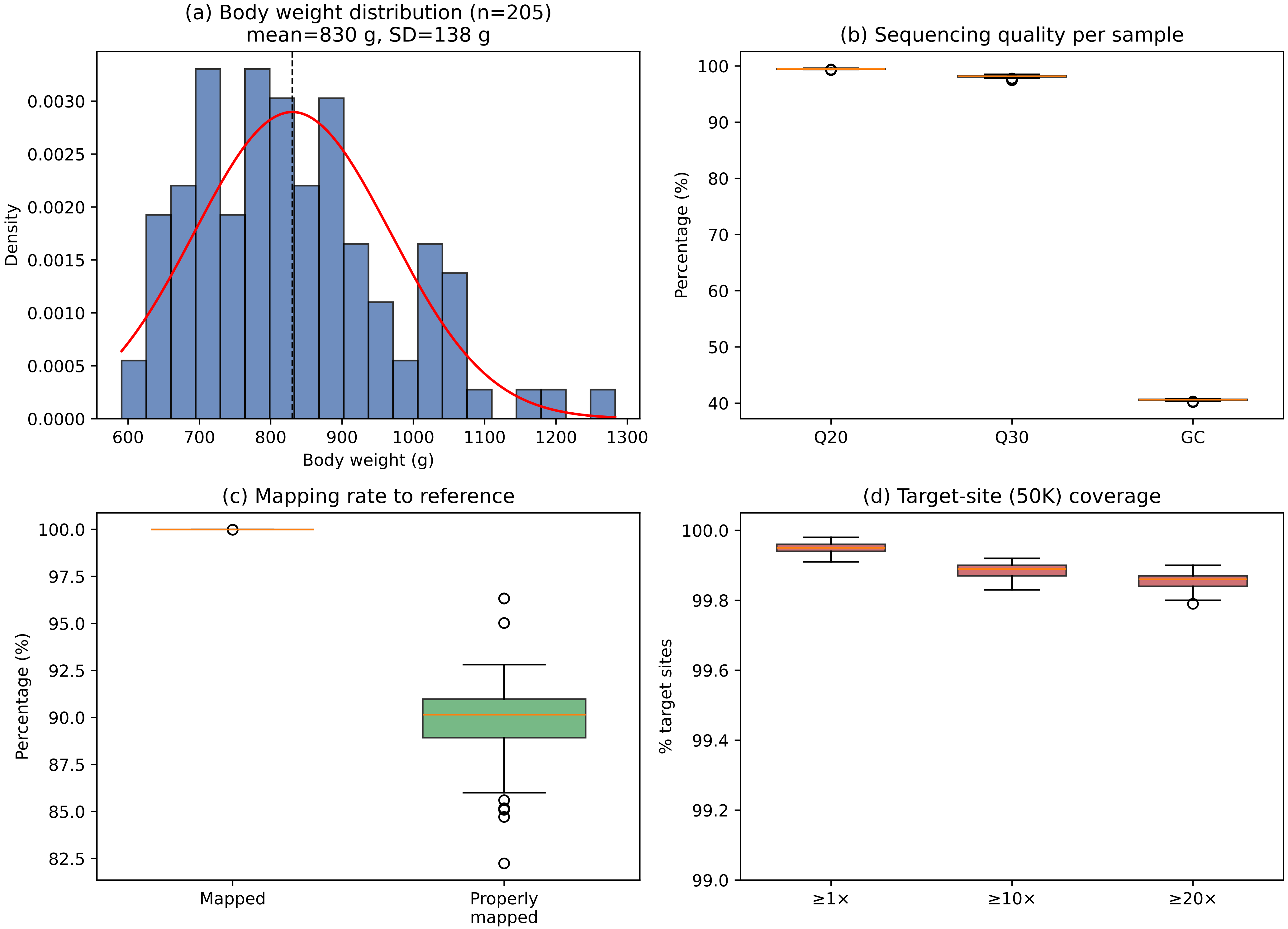

(a) Body weight distribution (n=205)
mean=830 g, SD=138 g
Density
0.0000
0.0005
0.0010
0.0015
0.0020
0.0025
0.0030
600
700
800
900
1000
1100
1200
1300
Body weight (g)
(b) Sequencing quality per sample
Percentage (%)
40
50
60
70
80
90
100
Q20
Q30
GC
(c) Mapping rate to reference
Percentage (%)
82.5
85.0
87.5
90.0
92.5
95.0
97.5
100.0
Mapped
Properly mapped
(d) Target-site (50K) coverage
% target sites
99.0
99.2
99.4
99.6
99.8
100.0
≥1×
≥10×
≥20×
