# Supplementary material for: A Genome-Wide Association Study Identifies Candidate SNPs and Genes Associated with Body Weight in the Northern Snakehead (Channa argus) Using a 50K SNP Array
Source: Animals (Basel). 2026 Aug 8;16(16):2471. doi: 10.3390/ani16162471 (PMC13508929; doi:10.3390/ani16162471)
Supplement: Supplementary file 1 [file animals-16-02471-s001.zip › FigureS2.pdf]

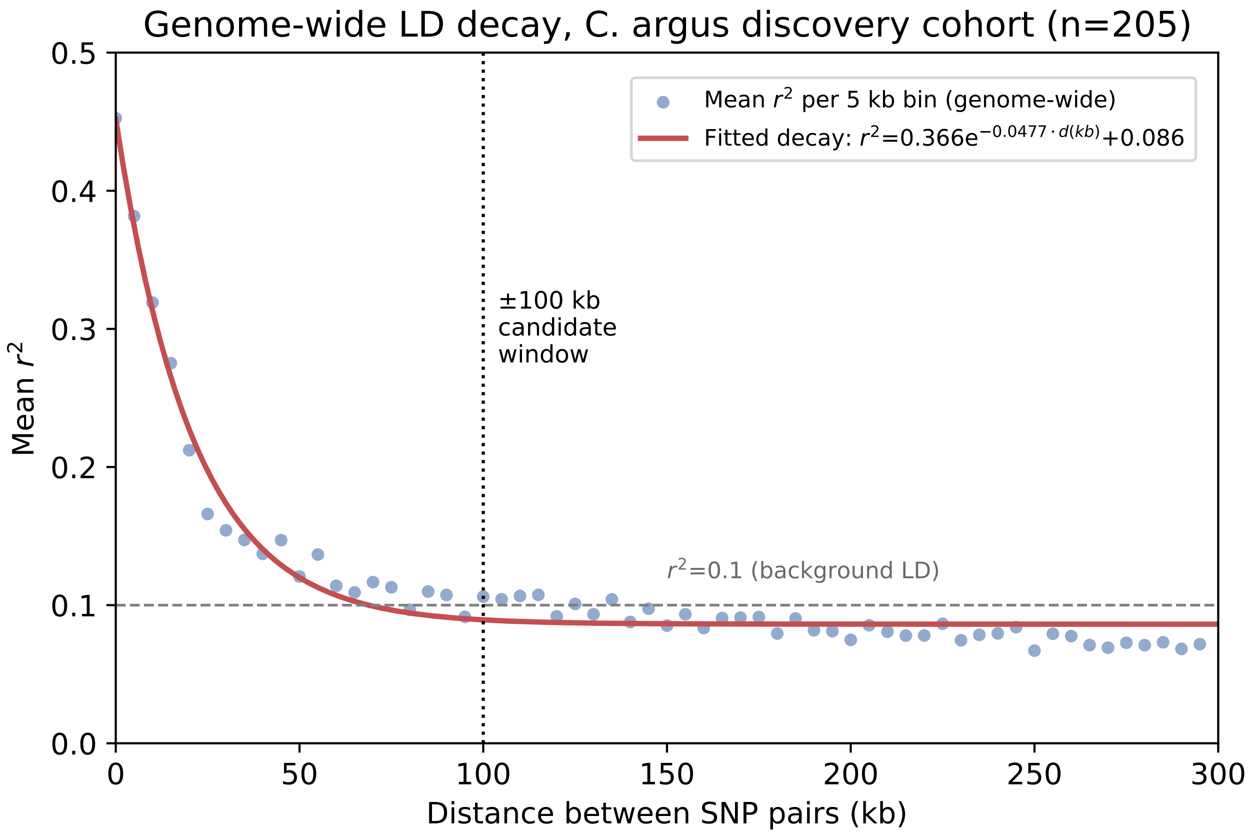
Genome-wide LD decay, C. argus discovery cohort (n=205)
Mean r^2 per 5 kb bin (genome-wide)
Fitted decay: r^2=0.366e$^{-0.0477 \cdot d(kb)}$+0.086
±100 kb
candidate
window
r^2=0.1 (background LD)
Mean r^2
Distance between SNP pairs (kb)
0.0
0.1
0.2
0.3
0.4
0.5
0
50
100
150
200
250
300
